# The Role of IRF9 Upregulation in Modulating Sensitivity to Olaparib and Platinum-Based Chemotherapies in Breast Cancer

**DOI:** 10.3390/genes15070959

**Published:** 2024-07-22

**Authors:** SeokGyeong Choi, Han-Gyu Bae, Dong-Gyu Jo, Woo-Young Kim

**Affiliations:** 1 College of Pharmacy, Sookmyung Women’s University, Seoul 04310, Republic of Korea; choi9174@gmail.com; 2 School of Pharmacy, Sungkyunkwan University, Suwon 16419, Republic of Korea; baehg159@gmail.com (H.-G.B.); jodg@skku.edu (D.-G.J.); 3 Muscle Physiome Research Center, Sookmyung Women’s University, Seoul 04310, Republic of Korea; 4 Drug Information Research Institute, Sookmyung Women’s University, Seoul 04310, Republic of Korea

**Keywords:** IRF9, PARP inhibitor, patins, resistance, biomarker

## Abstract

Poly(ADP-ribose) polymerase (PARP) inhibitors are targeted therapies that accumulate DNA damage by interfering with DNA repair mechanisms and are approved for treating several cancers with BRCA1/2 mutations. In this study, we utilized CRISPR-dCas9 interference screening to identify genes regulating sensitivity to PARP inhibitors in breast cancer cell lines. Our findings indicated that the interferon (IFN) signaling gene IRF9 was critically involved in modulating sensitivity to these inhibitors. We revealed that the loss of IRF9 leads to increased resistance to the PARP inhibitor in MDA-MB-468 cells, and a similar desensitization was observed in another breast cancer cell line, MDA-MB-231. Further analysis indicated that while the basal expression of IRF9 did not correlate with the response to the PARP inhibitor olaparib, its transcriptional induction was significantly associated with increased sensitivity to the DNA-damaging agent cisplatin in the NCI-60 cell line panel. This finding suggests a mechanistic link between IRF9 induction and cellular responses to DNA damage. Additionally, data from the METABRIC patient tissue study revealed a complex network of IFN-responsive gene expressions postchemotherapy, with seven upregulated genes, including IRF9, and three downregulated genes. These findings underscore the intricate role of IFN signaling in the cellular response to chemotherapy. Collectively, our CRISPR screening data and subsequent bioinformatic analyses suggest that IRF9 is a novel biomarker for sensitivity to DNA-damaging agents, such as olaparib and platinum-based chemotherapeutic agents. Our findings for IRF9 not only enhance our understanding of the genetic basis of drug sensitivity, but also elucidate the role of IRF9 as a critical effector within IFN signaling pathways, potentially influencing the association between the host immune system and chemotherapeutic efficacy.

## 1. Introduction

Poly(ADP-ribose) polymerase (PARP) inhibitors are damage-inducing targeted drugs currently approved by the U.S. Food and Drug Administration (FDA) for the treatment of ovarian, breast, prostate, and pancreatic cancers with BRCA1/2 mutations [1,2,3,4]. Among the 17 PARPs in humans, PARP inhibitors exert anticancer effects by targeting PARP1 [5]. PARP1 is a nuclear enzyme that plays a vital role in DNA repair, replication fork stabilization, transcription, inflammation, metabolism, and multiple other cellular processes [6]. Like most other targeted therapies, resistance to PARP inhibitors has been observed in many cases as the duration of drug use increases. Moreover, despite the promising preclinical discovery of synthetic lethality, not all patients with BRCA1/2 mutations respond to PARP inhibitors as expected [7]. Thus, research is actively underway to overcome this resistance and to identify biomarkers beyond BRCA1/2. Recent advances in RNAi and CRISPR gene-editing technologies have identified many biomarkers related to PARP inhibitor efficacy and have suggested novel therapeutic targets for new synthetic lethality strategies involving PARP inhibitors [8,9,10,11,12,13]. However, a biomarker with a better predictive value than BRCAness is not currently available for clinical use.

Type I interferon (type I IFN) was originally identified as a major defense mechanism against viral infections and is known to drive inflammation and immunosuppression in chronic infections [14,15]. Recently, type I IFN has attracted increasing attention for its direct and indirect antitumor effects. Many studies have demonstrated that type I IFNs have antitumor activity through the direct inhibition of tumor cell growth, as well as indirect effects, such as immunomodulation or the inhibition of angiogenesis [16,17,18,19]. Notably, DNA damage incurred during anticancer therapy activates the cyclic GMP-AMP synthase (cGAS)-stimulator of interferon genes (STING)-dependent interferon (IFN) response [20,21,22]. This activated IFN response interacts with the tumor microenvironment (TME), enhancing both innate and adaptive immune responses. This leads to more effective cancer treatment. However, IFN signaling activated by cytosolic DNA induced by DNA-damaging therapeutics may lead to cellular senescence [23,24] or apoptosis [25] through the cell’s own signaling pathways.

In this study, we conducted a CRISPR-dCas9 interference screen in a BRCA1/2-proficient breast cancer cell line to identify genes that modulate the cell response to the PARP inhibitor olaparib and identified IRF9 as a candidate. We hypothesized that type I IFN signaling regulates the sensitivity to DNA-damaging chemotherapies. The loss of the IRF/STAT family influenced the sensitivity to the PARP inhibitor in the triple-negative breast cancer (TNBC) cell lines. Furthermore, the correlation between the transcriptional increase in IFN signaling genes and DNA-damaging chemotherapy sensitivity (PARP inhibitor or Platins) was identified by analyzing data from the NCI Transcriptional Pharmacodynamics Workbench (NCI-TPW) [26] for the cell lines and from the METABRIC for clinical samples [27,28]. Our results demonstrated that the increased gene expression of IRF9, a member of the IRF/STAT family, serves as a biomarker for the responsiveness to these DNA-damaging chemotherapies and suggest that its suppression may be a mechanism by which cancer cells acquire PARP inhibitor resistance.

## 2. Materials and Methods

### 2.1. Cell Culture and Cell Line Constructions

MDA-MB-468 and MDA-MB-231 cells were cultured in RPMI or high-glucose DMEM containing 10% fetal bovine serum (Gibco, Grand Island, NY, USA), 100 units/mL penicillin, and 100 μL/mL streptomycin, respectively. All cells were maintained at 37 °C under 5% CO_2_ in an incubator. To generate the cell line stably expressing dCas9-KRAB (Addgene #85969) or dCas9-KRAB-MeCP2 (Addgene #122205), MDA-MB-468 or MDA-MB-231 cells were infected with lentiviruses expressing dCas9 and selected with blasticidin 2, 10 μg/mL respectively. Polybrene was added at a final concentration of 5 μg/mL to increase infecting efficiency.

### 2.2. Lentivirus Production

For lentivirus generation, HEK293T cells were transfected with these lentiviral vectors, packaging plasmids (psPAX2), and envelope-expressing plasmids (pMD2.G) using jetPRIME transfection reagents (Polyplus, Illkirch-Graffenstaden, France). Viral media were harvested about 68 h post-transfection, cleared via centrifugation, and then filtered with 0.45 μm. The aliquots were stored at −80 °C.

### 2.3. Genome-Scale CRISPRi Screening

The CRISPRi v2 top5 sgRNA library (Addgene #83969) was transduced into MDA-MB-468 dCas9-KRAB-expressing cells, with the percentage of transduced cells 5 days after transduction less than 30%. The library virus solution was treated for 24 h. Two days after transduction, the cells were selected with 1 μg/mL puromycin for 3 days. Cells were allowed to recover for 1 day in the absence of puromycin. From this point, a minimum 300× library coverage (3 × 10^7^ cells) was maintained for each group. The media containing olaparib and DMSO were changed every day for two weeks. Cells were harvested with a minimum 300× library coverage on day 14. Genomic DNA was isolated from frozen cell pellets and the sgRNA-encoding region was enriched, amplified, and processed for sequencing on Illumina Hiseq2500 50SE. Sequencing reads were aligned to the CRISPRi v2 library sequences and counted for each sgRNA sequence.

### 2.4. Clonogenic Survival Assays

MDA-MB-231/dCas9-KRAB-MeCP2 cells were infected with lentiviruses containing sgRNA (targeting IRF9, clonal constructed in this paper) or sgGFP-NT1 (Addgene #46914). MDA-MB-231 IRF9 TS and control cells were seeded in 6-well plates at a density of 200 cells per well. The adhered cells were treated for 14 days with olaparib, and medium with drug was changed on the 7th day. Cells were fixed with 4% paraformalin and methanol and then stained with crystal violet.

### 2.5. Quantitative RT-PCR Analysis

Total RNA was extracted from cells using Trizol (Invitrogen, Carlsbad, CA, USA) according to the manufacturer’s instructions, then cDNA was synthesized by iScript reverse transcriptase (Bio-Rad, Hercules, CA, USA). qRT-PCR analysis was performed with Power SYBR green (ThermoFisher Scientific, Waltham, MA, USA). The relative mRNA levels of IRF9 were normalized to the level of β-actin. Primers were IRF9-F; AGCTCTTCAGAACCGCCTAC, IRF9-R; CATGGCTCTCTTCCCAGAAA, β-actin-F; ATTGGCAATGAGCGGTTC, and β-actin-R; GGATGCCACAGGACTCCAT.

### 2.6. Western Blot Analysis

Cells were lysed with T-PER buffer (ThermoFisher Scientific) supplemented with protease and phosphatase inhibitor. Lysates were sonicated and centrifuged. Protein concentrations were determined using Pierce BCA Protein Assay kit (ThermoFisher Scientific). After boiling in the sample buffer, protein lysates were separated on SDS-PAGE and transferred to PVDF membranes. Membranes were blocked with 5% non-fat milk in TBST for 1 h at room temperature, followed by overnight incubation with primary antibodies in 5% BSA. Next, the membranes were incubated with corresponding HRP-conjugated secondary antibodies, the signals were detected using ECL substrate (ThermoFisher Scientific) on the Amersham Imager 680 (GE Healthcare Life Sciences, Marlborough, MA, USA). Antibodies used for immunoblotting analysis were IRF9; sc-365893, STAT2; sc-1668, α-tubulin; sc-23948 (Santa Cruz Biotechnology, Dallas, TX, USA).

### 2.7. Public Data Analysis

DepMap portal “https://depmap.org/portal/ (accessed on 13 June 2024)” was used to obtain cell line olaparib or carboplatin response data (PRISM Repurposing screen data) [29]. Clinical data were analyzed using the METABRIC, Nature 2012 and Nat Commun 2016 obtained from cBio Portal “https://www.cbioportal.org/ (accessed on 15 June 2024)” [27,28].

### 2.8. Statistical Analysis

All data were presented as mean ± standard deviation (SD), as indicated in the figure legends. GraphPad Prism 8 software (GraphPad Software, San Diego, CA, USA) was utilized for all statistical analyses. For comparisons between the two groups, statistical significance was evaluated using an unpaired t-test. Statistical significance was indicated as * *p* < 0.05, ** *p* < 0.01, *** *p* < 0.001.

## 3. Results

### 3.1. Transcriptional Suppression of IRF9 Induces PARP Inhibitor Resistance

**Figure 1:**
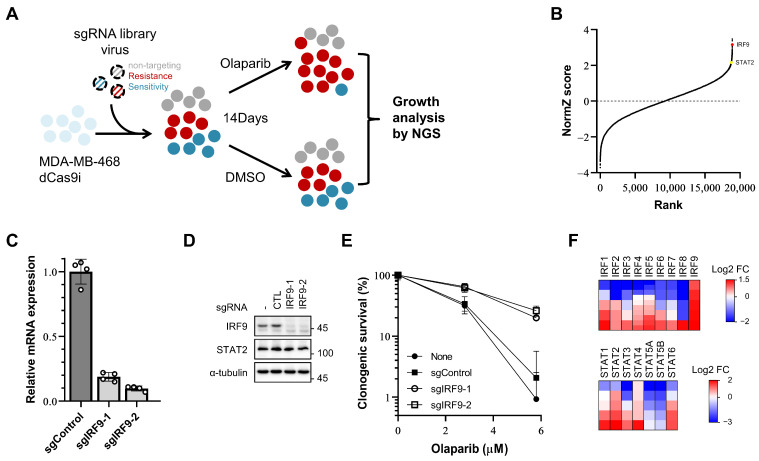
A CRISPR/dCas9 interference screen identifies IRF9 and other IRF/STAT family as resistance-inducing genes in TNBC cells. (**A**) Schematic of olaparib responsiveness screens. The response of cells to olaparib is represented by colors. Grey, neutrally responding; red, resistant; blue, sensitive. (**B**) Gene rank plots from dCas9-KRAB expressing MDA-MB-468 CRISPRi screens determined by comparing olaparib to DMSO vehicle treatment. Genes are ranked by the NormZ score derived from the DrugZ algorithm. IRF9 and STAT2 are marked in red and yellow, respectively. (**C**,**D**) qPCR analysis of IRF9 mRNA level and immunoblot analysis showing the IRF9 protein level in generated IRF9 TS MDA-MB-231 CRISPRi cell lines. (**E**) Clonogenic survival assay of IRF9 TS MDA-MB-231 cells. Cells were plated for clonogenic growth upon olaparib (2.8 and 5.8 μM) treatment. Data are presented as mean ± SD (*n* = 3). (**F**) Heatmap showing log2 fold change in sgRNA abundance. Each column represents one gene; each row represents one sgRNA. Five sgRNAs per gene except IRF4 and IRF5 (10 sgRNAs for these two).

To identify the genes whose repression affects the responsiveness of breast cancer cells to PARP inhibitors, we conducted a genome-wide CRISPR dCas9 interference screen in the TNBC cell line MDA-MB-468 in the presence of olaparib. Cells expressing dCas9-KRAB (CRISPRi) were infected with a hCRISPRi-v2 sgRNA library targeting 18,905 genes with 5 sgRNAs per gene [30]. These cells were then divided into treatment groups: one group receiving 1.5 µM olaparib (a PARP inhibitor) and the other group treated with DMSO (a vehicle). After 14 days, the surviving cells were harvested, the sgRNA was sequenced, and a bioinformatic analysis was conducted using the DrugZ algorithm (Figure 1A) [31].

Among the top hits, IRF9 was identified as the fourth gene that induced resistance when transcription was repressed by guide RNA and dCas9-KRAB (Figure 1B). IRF9 mediates the type I interferon response by forming IFN-stimulated gene (ISG) factor 3 (ISGF3), along with phosphorylated STAT1 and STAT2 (Y701 of human STAT1, Y690 of human STAT2) [14,32]. Surprisingly, STAT2, another component of ISGF3, also ranked high (144th out of 18,905 genes) (Figure 1B). The influence of IRF9 suppression on the PARP inhibitor responsiveness was further confirmed in another TNBC cell line, MDA-MB-231. We generated IRF9 transcription-suppressed (TS) MDA-MB-231 cell lines using two different sgRNAs (Figure 1C,D). In the clonogenic survival assays, these IRF9 TS cell lines exhibited increased resistance and survival rates (60% at 2.8 µM and 25% at 5.8 µM) compared to those of the control cells (30% at 2.8 µM and 1–2% at 5.8 µM) in the presence of olaparib (Figure 1E). These results confirm that the suppression of IRF9 leads to PARP inhibitor resistance in breast cancer cells in a way that intrinsically enhances cellular survival.

To determine whether similar intrinsic effects are present for other genes within the IRF/STAT family, we reanalyzed the read counts of sgRNAs for the 16 genes. Changes in drug responsiveness were assessed using log2-fold change values, focusing on cases where 80% or more of the sgRNAs used showed a consistent effect. Resistance was induced by IRF9, STAT2, and STAT4, and increased sensitivity was observed for IRF8, STAT5A, and STAT5B (Figure 1F).

**Figure 2:**
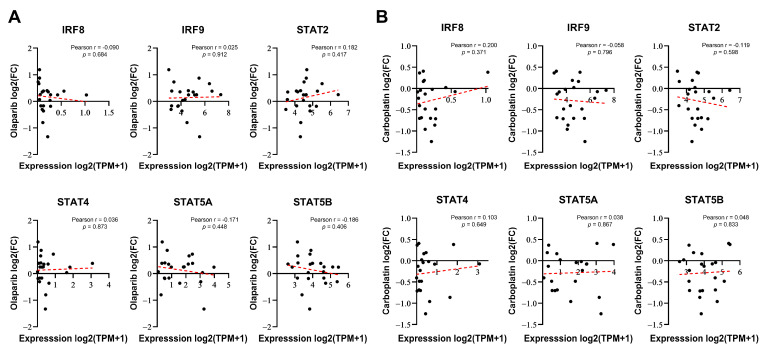
IRF/STAT family baseline expression does not correlate with sensitivity to olaparib or carboplatin in BRCA1/2 WT breast cancer cell lines. (**A**,**B**) Correlation analysis of each gene’s baseline expression versus olaparib sensitivity in 22 BRCA1/2 WT breast cancer cell lines, derived from DepMap Portal PRISM Repurposing screen data. Correlation analysis was conducted using Pearson correlation coefficient of gene expression versus olaparib or carboplatin responses (log2 fold change in abundance). Each dot represents a cell line, and the dashed red line indicates a linear regression line.

To determine whether the expression of certain genes correlates with the responsiveness of cells to DNA-damaging drugs, we considered two possibilities: intrinsically, the basal expression of these genes may be linked to resistance or, alternatively, their expression induced by DNA-damaging drugs could be responsible for resistance or sensitivity. Therefore, we investigated whether the basal expression levels of six genes selected from Figure 1F were correlated with the responsiveness to PARP inhibitors in breast cancer. We compared the responses to olaparib (log2-fold change in cell abundance) with baseline gene expression data from 22 BRCA1/2 WT breast cancer cell lines using PRISM Repurposing screen data from the DepMap portal [29]. None of these genes demonstrated a significant relationship with sensitivity to olaparib (Figure 2A). Similarly, no significant correlation was observed with carboplatin (Figure 2B), another anticancer drug that is known as a classic DNA-damaging agent. Although IRF8, IRF9, STAT2, STAT4, and STAT5A/B were identified as regulators of PARP inhibitor sensitivity in the screening, their basal expression levels did not correlate with drug responsiveness.

### 3.2. DNA-Damaging Agent-Induced IRF9 Upregulation Modulates the Drug Sensitivity of Cancer Cells In Vitro

**Figure 3:**
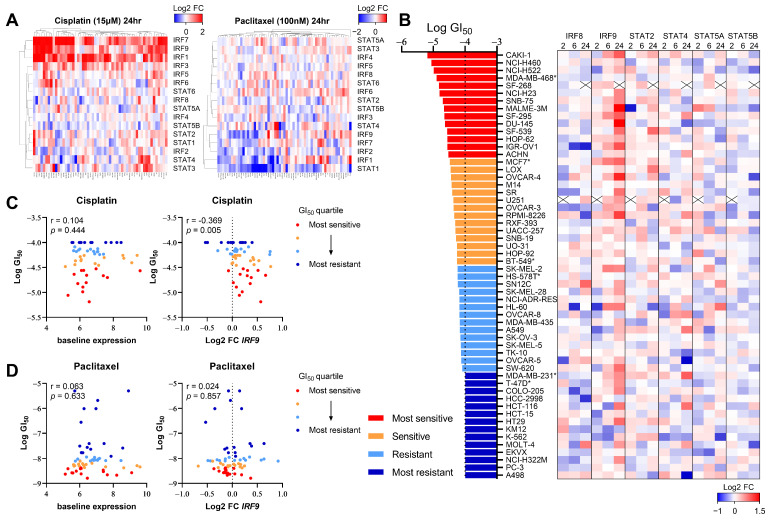
The upregulation of IRF9 expression by DNA-damaging chemotherapy correlates with drug sensitivity in various cancer types. (**A**) Heatmap showing log2 fold change in IRF/STAT family expression in NCI-60 cell line panel treated with cisplatin 15 μM (left) or paclitaxel 100 nM (right) for 24 h. Gene expression upregulation and downregulation are indicated by red and blue, respectively. (**B**) Heatmap showing log2 fold change in each gene expression in 56 human cancer cell lines treated with cisplatin 15 μM for 2 h, 6 h, or 24 h. Cell lines are ranked from the lowest to highest log GI50. A cross is shown where data were not available. *: breast cancer cell lines. (**C**,**D**) Dot plot showing baseline expression (left) and log2 fold change in IRF9 at 6 h (right) against log GI50 values for cisplatin or paclitaxel. Cell lines with available GI50data (cisplatin; *n*  =  56, paclitaxel; *n* = 60) were separated into quartiles, with the most sensitive (red) to most resistant (blue) shown. Pearson correlation coefficient and *p* value is shown.

Given that DNA damage induced by chemotherapy or radiation therapy (RT) activates interferon signaling [20,21,22,33], we investigated whether this change in the expression pattern correlated with the responsiveness to DNA-damaging drugs. We first assessed whether the transcription of IRF/STAT family genes was altered in human cancer cell lines based on the mechanism of the action of anticancer drugs. Using the NCI Transcriptional Pharmacodynamics Workbench (NCI-TPW) dataset [26], we compared transcriptomes over time following drug treatment. As anticipated, the transcript levels of IRF/STAT family genes in many cell lines changed 24 h after treatment with cisplatin, one of the most widely used DNA-damaging agents in clinical settings (Figure 3A). Specifically, IRF1, IRF7, and IRF9 levels tended to increase in many cell lines, and STAT5B, albeit to a lesser extent, mostly decreased after treatment. Conversely, no significant changes were observed with the treatment of cells with paclitaxel, a microtubule-stabilizing agent, except for a decrease in STAT1 (Figure 3A).

To determine whether the six genes identified as influencing the responsiveness to the PARP inhibitor in the CRISPR screen also correlated with cisplatin sensitivity, we analyzed the transcriptional changes of each gene over time in 56 cell lines, using the 50% growth inhibitory concentration (GI50) as a reference (Figure 3B). Notably, changes in IRF9 expression appeared to correlate with cisplatin sensitivity (Figure 3C); cell lines more sensitive to cisplatin, indicated by a lower GI50, exhibited an increased IRF9 expression post-treatment. However, the baseline expression levels of IRF9 were not associated with the GI50, as indicated in Figure 2. Furthermore, paclitaxel sensitivity showed no correlation with either the baseline or post-treatment changes in IRF9 expression (Figure 3D). These findings suggest that the upregulation of IRF9 following treatment with DNA-damaging agents is a common response across various tissues and is linked to drug sensitivity in a manner independent of immune cell involvement.

### 3.3. IRF9 Is Upregulated in Patients Who Receive Chemotherapy

**Figure 4:**
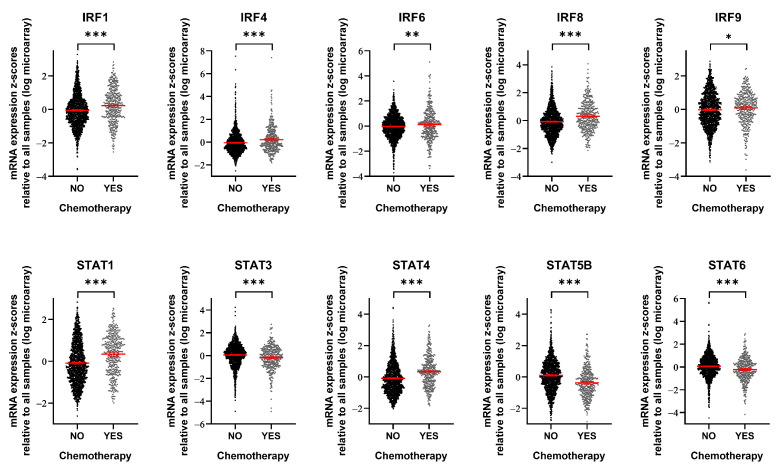
Breast cancer patients treated with chemotherapy show alterations in IRF/STAT family expression. mRNA expression level of IRF/STAT family in breast cancer patients who had been treated with chemotherapy or not. Dots represent each patient and are shown with the mean and 95% confidence interval (red line). Data were obtained from METABRIC, Nature 2012 and Nat Commun 2016 (No, *n* = 1568; YES, *n* = 412) [27,28]. * *p* < 0.05, ** *p* < 0.01, *** *p* < 0.001.

To assess the clinical relevance of our findings, we utilized METABRIC mRNA expression data [27,28] to compare the expression levels of specific genes in breast cancer patients with and without chemotherapy (including methotrexate, epirubicin, doxorubicin, and 5-fluorouracil) (Figure 4). Among the 16 IRF/STAT family genes we examined, 10 showed differential expression depending on chemotherapy exposure. IRF1, IRF4, IRF6, IRF8, IRF9, STAT1, and STAT4 were significantly upregulated following chemotherapy. In contrast, STAT3, STAT5B, and STAT6 were significantly downregulated. Interestingly, although IRF7 expression increased in many cell lines during acute interferon response activation by the cancer cells themselves (Figure 3A), no significant changes were observed in patient samples. Conversely, IRF1 and IRF9 expression levels showed increasing trends in both the acute cancer cell line dataset and patient samples (Figure 3A and Figure 4, respectively). STAT5B demonstrated an overall decrease after 24 h of cisplatin treatment in the cell line data (Figure 3A), and a similar decrease was observed in patient samples after chemotherapy (Figure 4). Collectively, these findings suggest that the expression of many IRF/STAT family genes, whose expression changed in cell lines, also changed in vivo after chemotherapy. These alterations likely have complex effects, influencing not only the interactions between cancer cells and their microenvironment, but also the survival signals within cancer cells.

## 4. Discussion

Type I IFNs are recognized for their benefits in cancer treatment, notably in promoting T-cell responses and preventing metastasis by interacting with the tumor environment. Type I IFNs are also integral to the DNA damage response in cancer cells. The impact of type I IFNs on cancer seems to depend on the intensity and duration of stimulation. DNA damage from anticancer treatments activates the cGAS-STING pathway, triggering a type I IFN response. Moreover, cancer cells often exhibit an increased expression of STAT1/2 and IRF9 for various reasons. Typically, ISGF3, a crucial transcription factor complex pivotal in the type I IFN response, is formed by phosphorylated STAT1, STAT2 (Y701 of human STAT1, Y690 of human STAT2), and IRF9. However, elevated expression levels lead to the formation of the U-ISGF3 complex without STAT1/2 tyrosine phosphorylation, altering the expression of some ISGs, including interferon-related DNA damage signature (IRDS) genes [34,35]. A strong and acute type I IFN response induces cytotoxic effects, such as growth arrest and apoptosis, and a weak and chronic response confers prosurvival benefits [32,34]. The cGAS-STING pathway also induces necroptosis by multiple mechanisms [36], demonstrating its anticancer role. It also induces autophagy, which may suppress cell death [37]. The cGAS-STING pathway produces a senescence-associated secretory phenotype (SASP) to increase senescence [38].

The effects and mechanisms of type I IFNs vary across cancer types, complicating the interpretation of their roles [32,34,35,39,40,41,42]. Our study identified IRF9 via a CRISPR screen aimed at identifying regulators of PARP inhibitor sensitivity in breast cancer. IRF9, the primary DNA-binding protein of ISGF3, has been shown to contribute to anticancer drug resistance (including cisplatin resistance) in colon cancer by upregulating IRDS genes [35,43]. Additionally, the expression levels of U-STAT1, U-STAT2, and IRF9 in small cell lung carcinoma (SCLC) cell lines correlate with survival after DNA-damaging chemotherapy. Cell lines with high levels of STAT1, STAT2, and IRF9 were resistant to DNA-damaging agents, and the knockdown of STAT1 or IRF9 (but not STAT2) restored cell sensitivity [35]. However, a comprehensive analysis across nine cancer types revealed no correlation between the baseline IRF9 expression and cisplatin sensitivity, but an increase in IRF9 expression following DNA-damaging chemotherapy correlated with drug responsiveness. Like this, in the SETUP (Sequential Evaluation of Tumors Undergoing Preoperative Chemotherapy) trial, TNBC patients with low tumor IRF9 expression during chemotherapy had a seven-fold higher rate of metastatic recurrence. This study also suggested that IRF9 is a marker for a T-cell-inflamed tumor [44]. These conflicting results highlight the variable impact depending on the cancer type and drug mechanism.

Among the IRF/STAT family members, IRF7 and STAT1, which are among the 49 IRDS genes [34], did not significantly affect the drug responsiveness according to the CRISPR screen. In breast cancer, however, the suppression of IRF9 or STAT2 transcription induces resistance to the PARP inhibitor. These findings suggest that genes increasing IRDS and others may contribute more to drug responsiveness than IRDS genes themselves. Additionally, the increase in IRF9 expression post-treatment was strongly correlated with drug responsiveness across nine cancer types, possibly due to cell death effects, such as apoptosis through the activation of caspase. In addition, studies have shown that IRF9 repressed SIRT1 expression in a variety of diseases and that the downregulation of SIRT1 attenuated deacetylase activity and activated p53, which enhanced the inflammatory response and apoptosis [45,46,47]. The anticancer roles of the cGAS-STING pathway through other mechanisms like the induction of necroptosis and senescence, as described earlier, may also contribute to the role of IRF9 that we identified.

In conclusion, this study underscores the significance of changes in IRF9 expression among various IRFs/STATs in relation to drug responsiveness, which is applicable across cancer types both in vitro and in vivo. The modulation of IRF9 expression after treatment with a PARP inhibitor or cisplatin has potential as a predictive biomarker. Moreover, the ectopic induction of IRF9 might enhance the efficacy of cisplatin. As anticancer strategies evolve, regulating the immune response remains a promising approach for new therapies. However, the complex interactions between the tumor immune environment and tumor cells continue to pose challenges in achieving the desired therapeutic effects. This research broadens our understanding of the complex role of type I IFN signaling in cancer by identifying IRF9 as a key regulator of therapeutic responses.

## Data Availability

The original contributions presented in the study are included in the article; further inquiries can be directed to the corresponding author.
